# Possible Involvement of Descending Monoaminergic Pathways in Colorectal Dysmotility Using a Rat Model of Colitis

**DOI:** 10.1111/nmo.70277

**Published:** 2026-02-24

**Authors:** Natsufu Yuki, Yuuno Hiroki, Tomoya Sawamura, Ayuna Mori, Kazuya Takashima, Yuuki Horii, Takahiko Shiina, Yasutake Shimizu

**Affiliations:** ^1^ Department of Basic Veterinary Science, Laboratory of Physiology, Joint Graduate School of Veterinary Sciences Gifu University Gifu Japan; ^2^ Department of Basic Veterinary Science, Laboratory of Physiology, Joint Department of Veterinary Medicine, Faculty of Applied Biological Sciences Gifu University Gifu Japan; ^3^ Institute for Glyco‐Core Research (iGCORE) Gifu University Gifu Japan; ^4^ Center for One Medicine Innovative Translational Research (COMIT), Institute for Advanced Study Gifu University Gifu Japan

**Keywords:** central nervous system, colitis, dopamine, gastrointestinal motility, serotonin, trinitrobenzenesulfonic acid, TRPV1 cation channels

## Abstract

**Background:**

Colonic inflammation is known to cause intestinal dysmotility. We examined the possible involvement of descending monoaminergic neurons projecting to the lumbosacral spinal cord in colorectal dysmotility using a rat model of colitis.

**Methods:**

Colitis was induced in rats by intracolonic administration of 2,4,6‐trinitrobenzenesulfonic acid. Motility in inflamed and noninflamed colorectal regions was assessed in vivo under anesthesia.

**Key Results:**

Colonic inflammation suppressed colorectal motility responses to noxious stimulus applied on inflamed colonic regions. The suppressed responses recovered as inflammation improved. In a subset of rats with colitis, basal motility in noninflamed regions was significantly enhanced, and this was abolished by intrathecal administration of serotonergic and dopaminergic receptor antagonists to the lumbosacral spinal cord. In some rats, enhanced basal motility spontaneously subsided then returned to a hyperactive state. The re‐enhanced basal motility was also suppressed by monoaminergic receptor antagonists, suggesting intermittent activity of the descending monoaminergic neurons.

**Conclusions and Inferences:**

This study suggested that persistent noxious input from an inflamed colon activates descending monoaminergic neurons, leading to enhanced basal motility in noninflamed regions. Our findings provide important insights into the pathophysiology of inflammation‐associated dysmotility.

## Summary

- Colonic inflammation impaired motor function at the inflamed site and altered the motility of noninflamed colorectal regions through central neural mechanisms.
- Descending serotonergic and dopaminergic pathways, which are normally involved in pain modulation, contribute to enhanced motility in noninflamed colorectal regions during colitis.
- Our findings shed light on a previously underappreciated central mechanism of dysmotility in colitis and provided important insights into the pathophysiology of defecation abnormalities caused by colitis.

## Graphical abstract

Colonic inflammation impaired motor function at the inflamed region and altered the motility of noninflamed colorectal regions through central neural mechanisms in rats. Descending serotonergic and dopaminergic pathways, which are normally involved in pain modulation, contribute to enhanced motility in noninflamed colorectal regions during colitis.

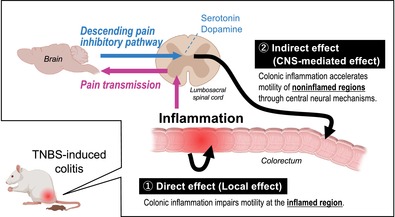

## 1 Introduction

Intestinal inflammation is known to cause intestinal dysmotility related to altered bowel habits [1, 2]. Experiments on various inflammatory model animals have shown indiscriminate impairment of a large number of enteric nervous system (ENS) neurons involved in cholinergic, adrenergic, purinergic, and/or nitrergic neurotransmission [3]. Moreover, inflammation impairs the function of enteroendocrine cells, such as 5‐hydroxytryptamine (5‐HT)‐containing enterochromaffin cells, which act as sensory transducers in the mucosa [4]. Furthermore, dysfunction of the interstitial cells of Cajal (ICC) was found in the inflamed intestine [5, 6]. In addition to intestinal dysmotility during acute inflammation, prolonged persistence of altered motility patterns has been commonly observed even after the inflammation has resolved [1, 7, 8, 9]. Persistent motility abnormalities are considered to be due to plastic changes in the ENS components or sustained hyperexcitability of enteric neurons in the post‐inflammatory phase [1, 2, 9, 10, 11]. Taken collectively, it would be reasonable to consider that intrinsic disorders of the regulatory mechanisms of the intestinal tract are primarily responsible for intestinal dysmotility during active inflammation and after resolution of the initiating inflammatory event.

Dysmotility associated with colitis has been thought to be mainly caused by abnormalities in the intrinsic mechanisms of the intestine, but the pathophysiological role of the central nervous system remains unclear. We have previously shown that noxious stimulation by intraluminal administration of capsaicin to the colorectum activates serotonergic and dopaminergic descending pathways from the brain to the spinal defecation center, thereby enhancing colorectal motility through the pelvic nerves [12, 13]. Based on the fact that the serotonergic and dopaminergic descending pathways are major components of descending pain inhibitory pathways, we proposed that abdominal pain causes defecation disorder through activation of descending monoaminergic neurons [14, 15]. Increased numbers of sensory fibers expressing the capsaicin receptor TRPV1 have been reported to be related to visceral hypersensitivity in patients with inflammatory bowel disease [16, 17, 18]. Considering our previous finding that TRPV1‐mediated signals were essential for activation of the descending pathways secondary to capsaicin administration to the colorectum [12], we hypothesized that excessive activation of descending pain inhibitory pathways would contribute to colorectal dysmotility and altered bowel habits.

The purpose of this study was to clarify the contribution of descending monoaminergic pathways to colorectal dysmotility in a rat model of colitis‐induced by 2,4,6‐trinitrobenzenesulfonic acid (TNBS), which has been reported to cause changes in colorectal motility [1, 19, 20]. Using an in vivo recording system, we evaluated time‐dependent changes in colorectal motor responses to intraluminal administration of capsaicin after induction of colitis in rats. In addition, we examined the effects of colitis on the motility of the colorectum outside the inflamed region.

## 2 Materials and Methods

### 2.1 Ethics Statement

All experimental procedures conformed to the “Regulations for Animal Experiments in Gifu University” and were approved by the president of the university after review by the Committee for Animal Research and Welfare of Gifu University (20,220,061, 20,230,179). The regulations of Gifu University conform to the Japanese “Act on Welfare and Management of Animals” and “Standards Relating to the Care and Keeping and Reducing Pain of Laboratory Animals (Notice of the Ministry of the Environment No. 88 of 2006).”

### 2.2 Animals

Male Sprague–Dawley rats (8–10 weeks old, 250–350 g) were purchased from Japan SLC (Shizuoka, Japan). The animals were housed in plastic cages at 22°C with a 12 h:12 h light–dark cycle (lights on at 07:00 to 19:00) and given free access to laboratory chow (MF; Oriental Yeast Co. Ltd., Tokyo, Japan) and water. Experimental procedures were performed according to the guidelines for the care and use of laboratory animals approved by the Committee for Animal Research and Welfare of Gifu University (20,220,061, 20,230,179).

### 2.3 Induction of Colitis by TNBS in Rats

The rats were fasted for 24 h with free access to water prior to colitis induction. Under light anesthesia with isoflurane inhalation, a flexible catheter was gently inserted 2–8 cm from the colorectum. TNBS solution at a dose of 5 mg dissolved in 40% ethanol to a total volume of 0.8 mL was slowly instilled through the catheter into the colorectum. Following administration, rats were maintained in a Trendelenburg position for 1 min to ensure uniform distribution of the solution and prevent leakage. Rats in the control group were given an enema of the same volume of saline instead of TNBS solution. Thereafter, the rats were returned to their original cages. Body weight, stool consistency, and presence of fecal blood were monitored daily.

### 2.4 Recording of Colorectal Motility In Vivo

Colorectal motility was recorded using an in vivo experimental system modified from that previously described [14]. Briefly, rats were anesthetized by intramuscular injection of ketamine hydrochloride (50 mg/kg) and injection of α‐chloralose (60 mg/kg) into the tail vein. The femoral artery was cannulated to simultaneously infuse anesthetics (ketamine hydrochloride at 3–5 mg/kg/h and α‐chloralose at 10–20 mg/kg/h) and measure blood pressure changes using a transducer. The colorectum was cannulated at the distal colon and anus and filled with saline solution warmed to 37°C from a Mariotte bottle connected to the distal colon cannula. Colorectal pressure was measured by a transducer connected to the anal cannula, and expelled fluid volume was measured by collecting fluid output through a one‐way valve onto a container connected to a force transducer. Baseline intraluminal pressure was maintained at 4–6 mmHg by adjusting the height of the Mariotte bottle. The bladder was also cannulated. After surgery, rats were kept for 1 h to stabilize colorectal motility and blood pressure. The in vivo recordings were performed using different rats on Days 2, 4, 7, and 14 post‐TNBS or saline injection. Specifically, experiments that involved inflamed regions comprised 5 rats in each group (control, Days 2, 4, 7, and 14). Rats that did not demonstrate diarrhea by Day 2 of post‐TNBS injection were excluded from the analysis. Experiments in noninflamed regions included 13 control rats and 23 TNBS‐treated rats. Experiments that involved monoamine receptor antagonists were conducted using eight TNBS‐treated rats that exhibited enhanced basal motility. Furthermore, the absence of visible erosion or ulceration at the recording site of the colorectum after euthanasia was macroscopically inspected to confirm that the measurements were obtained from noninflamed regions.

### 2.5 Drug Administration

Drug was administered into the lumbosacral spinal cord as described previously [14]. For administration of ketanserin (100 nmol), dolasetron (100 nmol), haloperidol (100 nmol), or 0.9% saline to the lumbosacral spinal cord, a 30‐gauge needle connected to a polyethylene tube was inserted on the dorsal surface between the L1 and L2 vertebrae, which correspond to L6–S1 spinal cord, until a tail flick or hind limb twitching was observed. For capsaicin administration, a catheter was placed in the colorectum. The catheter outlet was positioned at the oral end of the colorectum through the wall of the oral‐side cannula.

### 2.6 Reagents

The compounds used were isoflurane (VIATRIS, Canonsburg, PA, USA); α‐chloralose (Nacalai Tesque, Kyoto, Japan); ketamine hydrochloride (Daiichi Sankyo, Tokyo, Japan); ketanserin tartrate (AdipoGen, San Diego, CA, USA); dolasetron mesylate hydrate (Sigma–Aldrich, St. Louis, MO, USA); and haloperidol (Sumitomo Dainippon Pharma, Osaka, Japan). α‐chloralose was solubilized with 10% 2‐hydroxypropyl‐b‐cyclodextrin (FUJIFILM Wako Pure Chemical, Osaka, Japan) and then mixed with 0.9% saline for infusion. Capsaicin was dissolved in ethanol, then diluted with 10% Polyoxyethylene Sorbitan Monooleate (Tween 80; Tokyo Chemical, Tokyo, Japan) in saline to make a final ethanol concentration of 10%. Bicuculline and dolasetron were solubilized in distilled water and then diluted with 0.9% saline. Ketanserin was dissolved in dimethyl sulfoxide (DMSO; Nacalai Tesque) and then diluted with saline to make a final DMSO concentration of 10%. Haloperidol was diluted with 0.9% saline. TNBS (Fujifilm Wako Pure Chemical) was dissolved in 40% ethanol and used to induce colitis.

### 2.7 Data Analyses

**FIGURE 1:**
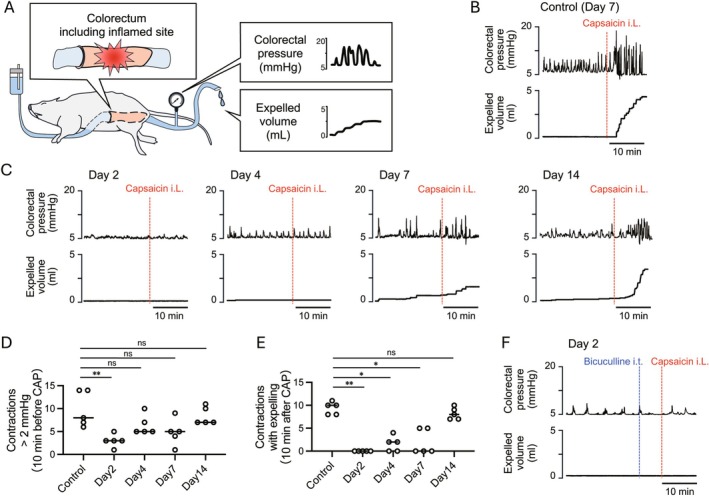
Effects of intraluminal administration of capsaicin on colorectal motility of the inflamed area of 2,4,6‐trinitrobenzenesulfonic acid (TNBS)‐induced colitis. (A) On the schematic outline of the in vivo recording of colorectal motility, the inflamed colorectal region is located between the cannulas at the distal colon and anus. (B) Representative traces of colorectal intraluminal pressure and expelled volume after intraluminal administration (i.L.) of capsaicin in control rats. (C) Representative traces of colorectal motility in rats on Days 2, 4, 7 and 14 after TNBS treatment. (D) Graph summarizing the number of contractions with an amplitude of > 2 mmHg before capsaicin administration in control and TNBS‐treated rats after 2, 4, 7, and 14 days (*n* = 5 per group). (E) Summarized graph shows the number of contractions with expelling after capsaicin administration in control rats and rats treated with TNBS after 2, 4, 7 and 14 days (*n* = 5 per group). (F) Representative traces show the effect of intrathecal administration (i.t.) of the GABA receptor antagonist bicuculline into the L6–S1 spinal cord on capsaicin‐induced colorectal motor response in rats at 2 days after TNBS treatment. ns, not significant; *p < 0.05; **p < 0.01.

**FIGURE 2:**
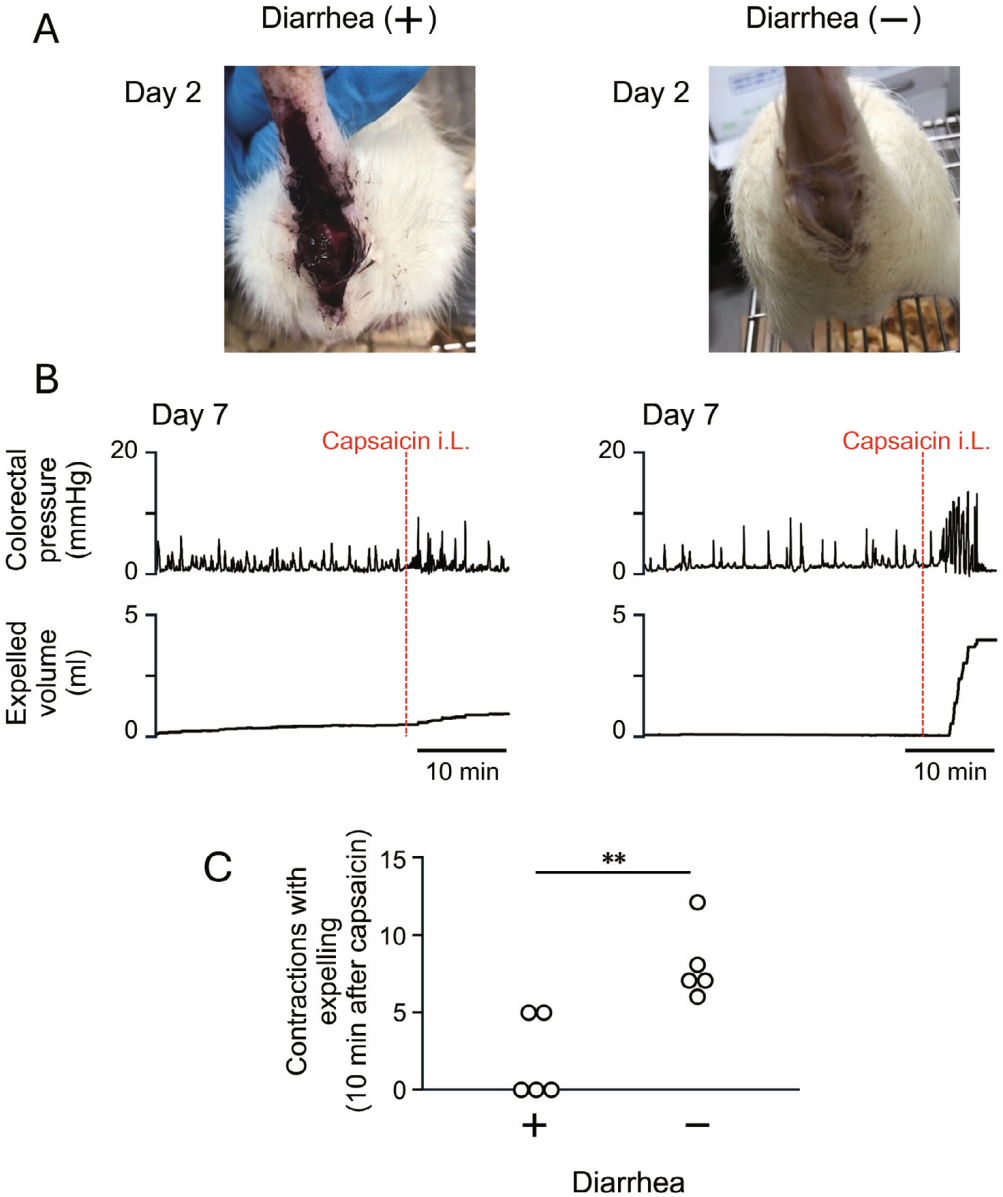
Relationship between inflammation severity and capsaicin‐induced colorectal motor responses in TNBS‐induced colitis. (A) Representative images of the perianal area of rats show the presence (left) and absence (right) of diarrhea at 2 days after TNBS treatment. (B) Representative traces of colorectal intraluminal pressure and expelled volume after intraluminal administration (i.L.) of capsaicin in rats with (left) and without (right) diarrhea. Motility is measured at 7 days after TNBS treatment. (C) Summarized graph shows the number of contractions with expelling after capsaicin administration in rats with and without diarrhea (*n* = 5 per group) **p < 0.01.

**FIGURE 3:**
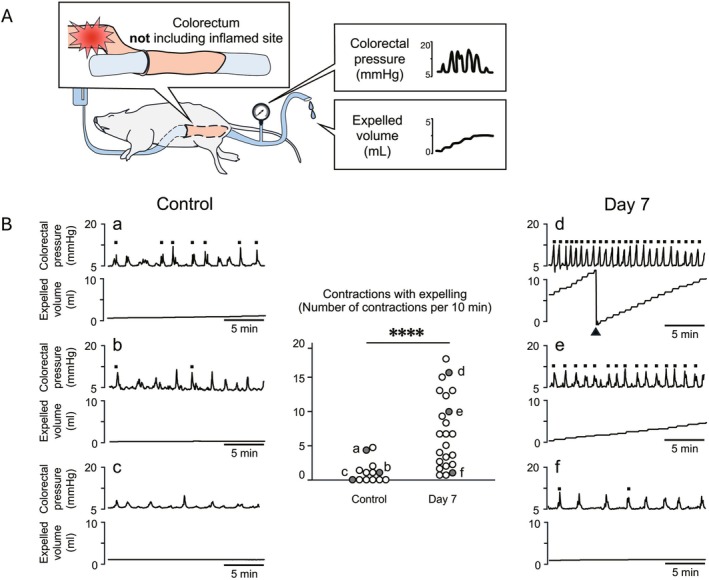
Basal motility of the noninflamed colorectal regions in rats with TNBS‐induced colitis. (A) Schematic outline of the experimental setup. TNBS is administered to a more proximal region of the colon, and motility is recorded from the colorectum, excluding the inflamed area. (B) Representative traces of colorectal intraluminal pressure and expelled volume from the distal colorectum in control (a–c) and TNBS‐treated (d–f) rats. Propulsive contractions are marked with black squares. The black triangle in panel (d) indicates the timing when the container for fluid output is manually emptied and reset. Summarized graph in the middle of panel (B) shows the number of contractions with expelling in the control (*n* = 13) and TNBS‐treated (*n* = 23) rats. The alphabetical symbols on the graph correspond to those indicated on the representative traces. ****p < 0.0001.

**FIGURE 4:**
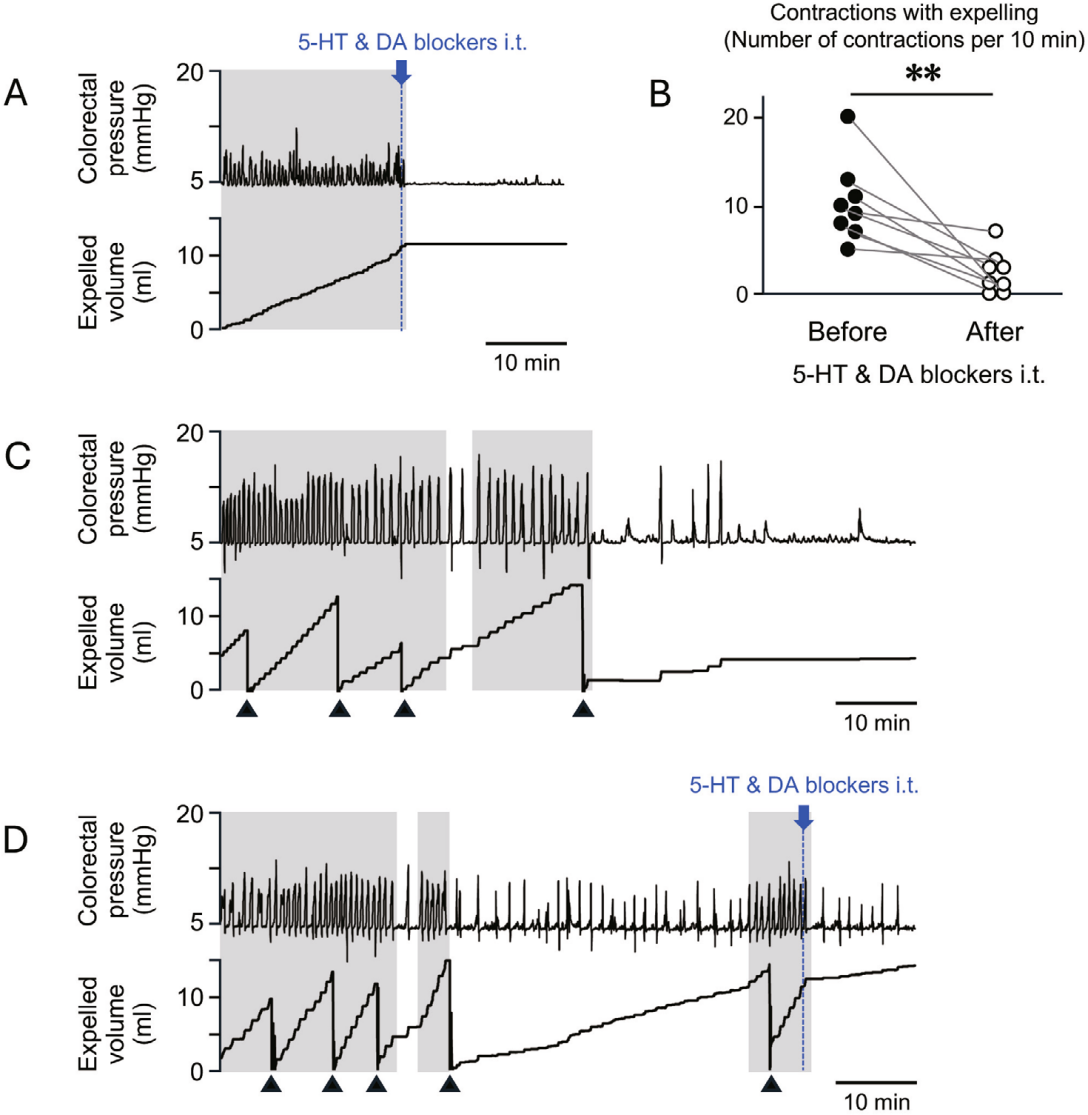
Effect of intrathecal administration of serotonergic and dopaminergic receptor antagonists on the basal motility of noninflamed distal colorectum in rats with TNBS‐induced colitis. (A) Representative traces of colorectal intraluminal pressure and expelled volume before and after intrathecal administration (i.t.) of monoaminergic receptor antagonists in a TNBS‐treated rat. Gray shaded areas indicate the duration with > 10 propulsive contractions every 10 min. (B) Summarized graph shows changes in the number of propulsive contractions before and after antagonist administration (*n* = 5). (C) Representative trace shows spontaneous reduction in basal motility without drug administration. (D) Representative trace shows spontaneous recurrence of enhanced basal motility and its suppression after intrathecal antagonist administration. The black triangles in panels (C, D) indicate the timing when the container for fluid output was manually emptied and reset. **p < 0.01.

Capsaicin‐induced colorectal motility was quantified using data from the initial 5 min after capsaicin administration. Contractions were counted when the intraluminal pressure increased by > 5 mmHg above baseline with accompanying intraluminal fluid expulsion. Data were presented as mean ± SD. For Figure 1D, multiple groups were compared using the Kruskal–Wallis test followed by Dunn's multiple comparisons test. For comparisons between two independent groups in Figures 2C and 3B, the Mann–Whitney *U* test was used. For Figure 4B, paired data before and after intrathecal drug administration were analyzed using the Wilcoxon signed‐rank test. All statistical tests were two‐sided, and *p* values < 0.05 were considered statistically significant. Statistical analyses were performed using GraphPad Prism version 10.5.0. Further details are available in the supplementary statistical analyses file.

## 3 Results

### 3.1 Effects of TNBS‐Induced Inflammation on Capsaicin‐Induced Colorectal Motor Response

Rats that had bloody diarrhea after TNBS treatment were anesthetized, and motility of the colorectum, including the inflamed part, was measured using an in vivo experimental setup (Figure 1A). Basal motility was defined as the number of contractions with an amplitude of > 2 mmHg. Following this criterion, basal motility was significantly suppressed on Day 2 post‐TNBS injection (Figure 1B–D). Intraluminal administration of capsaicin to the colorectum enhanced colorectal motility in control rats (*n* = 5), with a mean number of contractions with expelling of 9.4 ± 1.3 (Figure 1B). However, intracolonic capsaicin failed to induce colorectal motor response on Day 2 after TNBS treatment (0.0 ± 0.0, *n* = 5, *p* = 0.0001 vs. control; Figure 1C,E). Capsaicin‐induced colorectal motor response recovered in a time‐dependent manner; compared with the control, suppressed response almost totally recovered on Day 14 (Day 4: 1.6 ± 1.7, *n* = 5, *p* = 0.023; Day 7: 2.0 ± 2.7, *n* = 5, *p* = 0.012; Day 14: 9.0 ± 2.3, *n* = 5, *p* > 0.999; Figure 1C,E). The time course of response recovery corresponded to that of disappearance of gross mucosal lesions.

Given that GABAergic transmission within the spinal defecation center is known to suppress capsaicin‐induced colorectal motor responses [13, 21, 22, 23, 24], we then examined GABAergic inhibitory pathway activity during the acute phase of inflammation. As shown in Figure 1F, intrathecal administration of the GABA receptor antagonist bicuculline into the L6–S1 spinal cord did not restore capsaicin‐induced colorectal motor response.

### 3.2 Relationship Between Loss of Capsaicin‐Induced Colorectal Motor Response and Severity of Acute Colorectal Inflammation

After induction of colitis by TNBS treatment, diarrhea was effectively induced in some rats, whereas colonic inflammation signs, such as bloody stool around the anus, were not observed in some rats (Figure 2A). After capsaicin administration, colorectal motility was apparently enhanced in the rats that did not develop diarrhea (Figure 2B, right panel) but was strongly suppressed in the rats with diarrhea (Figure 2B, left panel). Quantitative analysis revealed significant differences in capsaicin‐induced responses between rats with and without diarrhea (2.0 ± 2.7 vs. 8.0 ± 2.3, respectively, *n* = 5, *p* = 0.008; Figure 2C).

### 3.3 Effects of TNBS‐Induced Inflammation on Motility of the Colorectum Outside the Inflamed Region

Next, we investigated the effects of colonic inflammation on the motility of the noninflamed colorectal region. The series of experiments showed that inflammation was induced at the more proximal part of the colon; recordings were made from the distal colorectum, which did not contain inflamed sites (Figure 3A). In our experimental setting, periodic fluctuation of colorectal pressure was observed under basal recording conditions. Of the 13 control rats, six showed increased colorectal pressure in the absence of expelled intraluminal liquid (Figure 3B‐c), whereas the remaining seven rats demonstrated some propulsive colorectal contractions, although the propulsive contractions were < 5 times every 10 min (Figure 3B‐a,b). Overall, although the baseline intraluminal pressure was set to the same level in both groups, the number of basal contractions every 10 min in the noninflamed colorectal region was significantly higher in rats with TNBS‐induced colitis (*n* = 23) than in the control rats (*n* = 13) (7.0 ± 5.3 vs. 1.2 ± 1.6, *p* < 0.0001; Figure 3B). Enhanced basal motility was defined as > 5 contractions with expelling every 10 min. Among the 23 rats with TNBS‐induced colitis, basal motility was enhanced in 13 rats (Figure 3B‐d,e), whereas the remaining 10 rats exhibited basal activity comparable with that in control rats (Figure 3B‐f).

### 3.4 Involvement of Serotonergic and Dopaminergic Transmission Within the Spinal Defecation Center in the Enhanced Basal Motility of the Noninflamed Colorectal Region in Rats With TNBS‐Induced Colitis

To investigate whether enhanced basal motility of the noninflamed colorectal region was due to continuous activation of serotonergic and dopaminergic descending neurons, serotonergic receptor antagonists (dolasetron and ketanserin) and the dopaminergic receptor antagonist haloperidol were intrathecally injected into the spinal defecation center while basal activity was increased. Intrathecal administration of monoaminergic receptor antagonists did not affect periodic contractions in normal rats without inflammation (data not shown) but suppressed the enhanced basal motility in rats with colitis (Figure 4A). Gray background in Figure 4 represents periods of contractions with expelling at a rate > 10 times per 10 min. Quantitative analysis revealed significant suppressive effects of these antagonists (before: 10.4 ± 4.6; after: 2.4 ± 2.4, *n* = 8, *p* = 0.008; Figure 4B).

Figure 4C represents a typical recording of enhanced basal motility, which spontaneously subsided in the absence of antagonist administration. Notably, in some cases, the spontaneously subsided motility returned to a hyperactive state despite the absence of stimulus; this recurrent enhanced basal motility was suppressed by intrathecal administration of monoaminergic receptor antagonists (Figure 4D).

## 4 Discussion

In this study, we examined the contribution of descending monoaminergic pathways to colitis‐induced colorectal dysmotility. Our findings indicated that severe inflammation in the colon disturbed colorectal motility responses to noxious stimulus. This was not surprising, because previous studies demonstrated that inflammation damages ENS neurons, enteroendocrine cells, and ICC, resulting in loss of motor function of the inflamed colon [4, 5, 6]. More importantly, we provided evidence that motility of the noninflamed colorectal region can be affected by the presence of inflammation in a different region of the colon. We observed that basal motility was typically enhanced at the noninflamed region. The enhanced basal motility was likely due to activation of descending monoaminergic neurons, which activate the spinal defecation center through release of 5‐HT and dopamine. To the best of our knowledge, this was the first study to show that the central neural pathway contributes to colorectal dysmotility during colitis.

The negative impact of inflammation on intestinal motility can be mainly attributed to motor function disorders. In this study, basal motility and capsaicin‐induced motility responses were suppressed when severe inflammation was induced by TNBS treatment. We have previously shown that increased activity of GABAergic neurons in some situations, such as chronic female sex hormone treatment and destruction of dopaminergic neurons in the substantia nigra in a Parkinson's disease model, resulted in loss of capsaicin‐induced colorectal motor response in rats [21, 25]. Therefore, we initially thought that the lack of capsaicin‐induced response in TNBS‐colitis rats in this study was increased activity of GABAergic neurons in the presence of colonic inflammation. However, administration of a GABA receptor antagonist did not allow recovery of colorectal responses to capsaicin. Therefore, we considered that colorectal damage was directly related to dysmotility during acute colitis, based on our findings of a correlation between loss of responses and colitis severity and the recovery of responses as the experimentally induced colitis recovered. Similarly, other studies demonstrated impaired motility in isolated intestinal preparations [6, 26]. Taken together, it would be reasonable to consider that the loss of motility response to intracolonic noxious stimulus on the inflamed regions is related to peripheral damages rather than functional changes in central regulatory mechanisms.

The most important finding of this study was that the presence of colonic inflammation accelerated the motility of noninflamed colorectal regions through mediation of the descending monoaminergic pathways. In our in vivo recording experiments, we sometimes encountered frequent contractions accompanied by expulsion of intraluminal liquid immediately after surgery. However, after keeping the rats for 1 h without treatment, colorectal motility was stabilized and the number of propulsive contractions was < 5 times every 10 min. The frequent propulsive contractions in the noninflamed region in rats with TNBS‐induced colitis even after stabilization allowed us to assume that the descending monoaminergic pathways operated under a basal state. Consistent with this assumption, enhanced basal motility was suppressed by intrathecal administration of serotonergic and dopaminergic receptor antagonists. Therefore, we propose a novel concept that the presence of inflammation in a specific site of the colon may affect motility of the noninflamed regions by persistent activation of descending monoaminergic pathways.

Persistent activation of descending monoaminergic pathways would be related to hyperalgesia, which is commonly promoted in an inflamed colon [1, 19, 20]. Descending monoaminergic pathways are known to act as a pain modulatory system, which is activated in response to noxious signals transmitted to the brain [27, 28]. In fact, we have previously reported that intracolonic administration of capsaicin as a noxious stimulus activated monoaminergic neurons descending from the brainstem to the lumbosacral spinal cord, resulting in increased colorectal motility [14]. Importantly, the effect of capsaicin in promoting colorectal motility is TRPV1‐dependent, because capsaicin failed to enhance motility in TRPV1‐knockout mice [12]. In a rat model of TNBS‐induced colitis, TRPV1‐expressing nerves were shown to be constitutively activated [18]. Accordingly, constitutive inputs from TRPV1‐mediated signals, which may be related to hyperalgesia, probably lead to persistent activation of descending monoaminergic pathways, causing increased basal motility at the noninflamed colorectal regions.

There were considerable differences in basal colorectal motility among the rats that showed bloody diarrhea. The variations could be explained by assuming intermittent monoamine release from descending pathways in the spinal cord, based on our findings that enhanced basal motility spontaneously resolved but recurred due to the action of the descending pathways after some time. Based on this assumption, enhanced basal motility can be recorded during experiments if rats are in the active phase of monoaminergic transmission, and vice versa. Sustained high‐frequency stimulation of dopaminergic neurons was reported to decrease the dopamine release, but this recovered after a substantial period of rest [29]. Continuous stimulation of serotonergic neurons of the raphe nuclei was demonstrated to deplete intracellular pools, which were replenished during rest [30]. Therefore, it is plausible that persistent nociceptive signaling from the inflamed site combined with repeated cycles of monoamine depletion and replenishment in the descending pathways contributes to intermittent enhancement of colorectal motility.

This study has several limitations that warrant discussion. First, all in vivo recordings were performed under anesthesia to stabilize physiological parameters. Consequently, in the awake state, the extent to which the enhanced basal motility observed in the noninflamed regions actually contributes to altered bowel habits, including diarrhea, remains unclear. Second, colonic dysmotility may persist for a long period even after resolving inflammation; however, we did not observe such long‐term abnormalities in the present study, in which the impaired capsaicin‐induced motor responses returned to normal by Day 14 in parallel with mucosal healing. This indicates that the colitis model generated by a single dose of TNBS used in this study primarily represents the pathophysiology of the acute inflammatory phase rather than that of the chronic phase. Thus, more complex pathological mechanisms are required to induce post‐inflammatory dysmotility. Future studies that use awake animal models and explore additional factors are warranted to fully identify the association between visceral pain, descending control, and the transition to chronic bowel dysfunction.

## 5 Conclusions

This study demonstrated that colonic inflammation impaired motor function at the inflamed site and altered the motility of noninflamed colorectal regions through central neural mechanisms. Specifically, we provided novel evidence that descending serotonergic and dopaminergic pathways, which are normally involved in pain modulation, contribute to enhanced motility in noninflamed colorectal regions during colitis. These findings shed light on a previously underappreciated central mechanism of dysmotility in colitis and provide important insights into the pathophysiology of defecation abnormalities caused by colitis.

## Author Contributions

Natsufu Yuki contributed to the collection, analysis, and interpretation of data and wrote the article. Yuuno Hiroki, Tomoya Sawamura, Ayuna Mori, Kazuya Takashima, and Yuuki Horii contributed to the collection, analysis, and interpretation of data. Takahiko Shiina participated in the design of the study and helped to draft the manuscript. Yasutake Shimizu was a supervisor in this study and revised the article critically for important intellectual content. All authors have read and approved the final version of this manuscript and agree to be responsible for all aspects of this work in ensuring that any questions relating to the accuracy or completeness of any part of this work are properly investigated and resolved. All persons designated as authors are qualified to be authors and all persons listed as qualified authors.

## Funding

This work was supported by Grants‐in‐Aid for Scientific Research (KAKENHI) from the Ministry of Education, Culture, Sports, Science, and Technology of Japan [Research Project Number: 23H00360] and Grant‐in‐Aid for JSPS Fellows from the Ministry of Education, Culture, Sports, Science, and Technology of Japan [Research Project Number: 24KJ1212].

## Conflicts of Interest

The authors declare no conflicts of interest.

## Data Availability

The data that support the findings of this study are available from the corresponding author upon reasonable request.
